# Drivers and barriers to sustained use of Blair ventilated improved pit latrine after nearly four decades in rural Zimbabwe

**DOI:** 10.1371/journal.pone.0265077

**Published:** 2022-04-01

**Authors:** Artwell Kanda, Esper Jacobeth Ncube, Kuku Voyi

**Affiliations:** Faculty of Health Sciences, School of Health Systems and Public Health, University of Pretoria, Pretoria, South Africa; Cranfield University, UNITED KINGDOM

## Abstract

**Background:**

Some latrines remain unused even under conditions of high coverage in rural areas of low- and middle-income countries. Not much is known on household latrine use in the long term in the absence of an intervention. The current work assesses drivers and barriers to sustained use of a ventilated improved pit latrine (Blair VIP) design where it originated and how rural households adapt it to climate change.

**Methods:**

A mixed methods study was conducted from November 2020 to May 2021 among rural households of Mbire district, Zimbabwe. A cross sectional survey of 238 households with Blair ventilated improved pit (BVIP) latrines was conducted using a questionnaire and a latrine observation checklist. Data were analysed using logistic regression. Qualitative data were collected using six focus groups among house heads and analysed by thematic analysis.

**Result:**

The latrine has perceived health, non-health and hygiene benefits for its sustained use. However, there are design, environmental and social barriers. The quantitative study indicated that determinants of latrine use were contextual (individual and household levels) and technology (individual level) factors. Focus groups indicated that latrine use was influenced by social, technology and contextual factors at multiple level factors. Interplay of factors influenced the intention to adapt the BVIP latrine to climate change. Local climate change adaptation strategies for the latrine were odour and erosion control, construction of the conventional latrine design and raised structures.

**Conclusion:**

The conventional BVIP latrine design is durable and relatively resilient to climate change with high local household use. High construction cost of the latrine causes households to build incomplete and poor quality designs which affect odour and fly control. These are barriers to sustained latrine use. The government should implement the new sanitation policy which considers alternative sanitation options and offer community support for adapting sanitation to climate change.

## Introduction

The global use of safely managed sanitation services in rural areas showed an increase of 1.48 percentage points/year from 2015–2020 at the national level, and by 2020 about 3.6 billion people still lacked safely managed services [1]. Climate change threatens efforts to serve them [2], potentially worsening the sanitation challenge. Even in areas of high sanitation coverage, latrine use was reported to be low [3, 4] indicating that the presence of a latrine does not translate into use. Sanitation coverage refers to the percent proportion of a population using improved sanitation facilities [5].

Most reports of research done on the use of latrines in rural communities of low- and middle-income countries (LMICs) are impact evaluation studies of interventions which use various sanitation options in different settings. They were reportedly done some months to a few years following the intervention end line [6–10]. Such evaluations commonly report behaviour change in the short term [11]. This could be because behaviour change is difficult to initiate and sustain [12], or that self-reported initial and long-term behaviour change may be difficult to identify. There is no standard approach to evaluate post-intervention latrine use. Further, the reliability of the methods used to assess latrine use is uncertain [7]. This could have led to variations in intervention follow-up times in latrine use impact studies, complicating the definition of sustained use. In this work, sustained use refers to the continued use of a sanitation facility at least six months post the intervention period [13].

Some factors which influence sustained use of latrines in rural communities of LMICs reported in literature were based on individual, household, community, technology (latrine) and socio-economic levels. Technology factors included the quality and completion of construction, type, functionality and age of a latrine [14, 15]. Individual-level perceived benefits of using a latrine were safety, security, privacy and convenience [16]. The availability of water also underpinned the use of water-borne sanitation options [14]. Local culture, beliefs and attitudes were reported to influence latrine use behaviour [16]. The educational level, age, gender and occupation of a house head influence latrine use [17, 18]. Household-level factors were household size and wealth [15]. These were follow-up studies to interventions with different packages and strategies. Despite the reported evidence of improved sanitation services, barriers that influence sustained use of various options remain unclear [13, 16, 19]. An understanding of factors which influence latrine use is important to inform future sanitation practice.

The Blair Ventilated Improved Pit (BVIP) latrine is a dry non-sewer on-site sanitation facility. It is a Zimbabwean innovation of the 1970s (named after Dr. Dyson Blair, former secretary, ministry of health) which got international recognition resulting in many current versions of the ventilated improved latrine [20]. The BVIP latrine later became known as the Ventilated improved pit (VIP) latrine globally. The conventional design comprises a brick-lined pit, concrete slab with a squat hole, PVC vent pipe, fly screen and brick-built superstructure with a roof to give a semi dark interior [21, 22]. A vent pipe offers odour control and a fly screen traps flies. The upgradable version of the latrine maintains the basic brick-lined pit and concrete slab design of the BVIP latrine with the superstructure built in stages [22]. Zimbabwe encourages the construction of a local sanitation technology innovation, the BVIP latrine for rural households. The country’s sanitation policy draft of 2017 which considers alternative options seems to have ended with pilot studies [e. g. 23] as the BVIP latrine remained the encouraged design in practice.

The sustainability and performance of sanitation technologies are subject to climate change whose potential impact on health outcomes is on the global research agenda [24]. Climate change impacts include environmental contamination, groundwater quality impairment, public health risks [2, 25], infrastructural damage, and floatation of faecal matter in pit latrines [25, 26]. Floods damage latrines especially those on loose soil, fill up pits with water and erode soil. They may leave households without permanent sanitation infrastructure and influence latrine use of damaged facilities. In 2015, the Mbire district civil protection department indicated that floods left 60% of the water and sanitation infrastructure destroyed which triggered the outbreak of cholera and typhoid [27]. While the use of the BVIP latrine may not be affected by unavailability of water during drought periods (except for handwashing), high air temperature during the summer period (up to 40°C) in this semi-arid area may influence latrine use.

Climate change has been linked to increased potential risk of diarrhoeal diseases [25, 26]. However, some of the perceived benefits of adapting sanitation to climate change include rationalising the choice of sanitation technologies to be used and unbundling of the sanitation options basket by adopting widely acceptable alternatives [25]. Climate change adaptation refers to accustoming in natural or human systems in response to actual or predicted /expected climatic hazards to prevent or reduce harm, or exploit opportunities [28]. Adaptation strategies to climate change may be hard/soft, reactive/proactive or effect-/cause-oriented [29]. An understanding of the factors which influence the use of a single sanitation option in different environmental settings in areas prone to climate change impacts may be useful to inform the selection of alternative options as an adaptation strategy.

Currently, there is no assessment report of long-term (over four decades) use of a single latrine option, as a nationally encouraged sanitation option, by households in poor rural communities vulnerable to climate change hazards in LMICs. Locally, no report has been given for the factors which influence the use of the BVIP latrine in under such settings. The research questions for this work were: (i) what are the factors which influence sustained use of the BVIP latrine in rural communities under low- and middle-income settings (LMISs) prone to climate change hazards, and (ii) how do households living under such conditions adapt the BVIP latrine to climate change? The current work reports a study conducted where no recent targeted intervention had been done. It is assumed to represent a long-term sanitation practice among rural households for over four decades of technology implementation but with low sanitation coverage (~35%). It is centred on the conventional BVIP latrine design because it appears there is no reported empirical evidence of the adoption of its upgradable versions outside pilot studies in Zimbabwe. Investigating factors which influence latrine adoption were not part of this work. However, latrine construction was discussed only as a factor which influences use.

The theoretical framework of the quantitative study was the integrated behavioural model for water, sanitation and hygiene [30] to categorise determinants of latrine use. It composes contextual, psychosocial and technology factors, each with five levels (S1 Table). The framework appears widely used to provide a methodology to analyse multiple levels of influences [13, 16, 19].

## Materials and methods

### Study design and area

A mixed methods research design comprising a cross sectional survey among randomly selected rural households and focus group participants sampled by snowballing was used for the study conducted in Mbire district found in Mashonaland Central Province, Zimbabwe. Details of the study area of this work were described elsewhere [31]. The district was purposively selected. It is mostly rural, and according to a national vulnerability assessment report [32] it represents a worst case scenario of poverty with low sanitation coverage. Understanding a worst-case scenario provides a baseline condition that allows focusing on conditions that need change, how change may be achieved and transferred to other scenarios. Mbire district is semi-arid, experiences high air temperature (40°C) in summer, low annual rainfall (450–650 mm), droughts and floods, particularly further north in the lower middle Zambezi valley. It is representative of how poor households with low access to sanitation services, use the BVIP latrine, adapt their sanitation needs to it even in the face of climate change, and use some climate change adaptation strategies to access their latrines.

### Sample size and selection of participants

The current work is part of an on-going study where the selection and recruitment of wards, villages and households, and determination of the sample size were published elsewhere [31]. The single population proportion formula [33] was used in a multistage sampling strategy to determine a sample size of 790 households which was used in earlier work. For this particular study, all households with BVIP latrines (238; 30.1%) were selected from the calculated sample size of 790 households. Briefly, five rural wards from the district, five villages from each ward, and households in a village were selected by simple random sampling (lottery method). Numbers of all the wards in the district were written on small pieces of paper and five were picked from a container one at a time without looking at them. This was repeated for villages in a ward for the five randomly selected wards. Proportional to size allocation was used to determine ward and village samples. The number of sample units to select from each stratum was made proportional to the number of sample units (households) within each stratum. In this case, the ward and village were separately treated as strata. A ward sample was determined as: number of households in that ward divided by the sum of households in the selected five wards, multiplied by the calculated study sample size. This was done for all the five wards and the five villages. At village level the actual households were selected by simple random sampling using a list of households in a village. A rural household where consent to participate was given was included. Abandoned households were excluded and replaced by the next eligible one. The target interviewee to participate in the questionnaire interview at the household was the female house head. If she was not available, then the male house head was recruited. The candidate participant was to be above 18 years of age, not mentally challenged and should have resided at the homestead for more than six months.

Participants for each focus group were adult (> 18 years of age) house heads (male and female) who were sampled by snowballing through village health workers in a village. Those who volunteered to participate by completing consent forms were invited. Selection was based on assumed knowledge in household sanitation indicated by participation in similar work before. Nine participants were invited for each focus group allowing for poor turnout. A heterogeneous group based on sex was used to allow a balanced discussion. Participants shared some previous knowledge and experience that allowed some degree of homogeneity. The focus group comprised male and female participants to allow for some (common male-female) tension that may serve to uncover deeper insights [34] into household sanitation issues.

Data collectors were local personnel from the ministry of health responsible for rural sanitation. It was assumed that they would remove the language barrier and do data collection as part of their routine work. This made it possible for them to do data collection by unannounced household visits to avoid the interviewee being aware beforehand. They were professionally trained in the design, operation, maintenance and use of the BVIP latrine. Further, they had experience in working with communities and project implementers in rural sanitation issues. However, it was impossible to blind them in the field. Data collectors had a 2-day training which ended with pre-testing the research instruments. To help reduce researcher bias some data were collected through the questionnaire, FGD and an observation checklist. Pre-field training with data collectors and regular field debriefing sessions help reduce bias [35].

### Variables, data collection and analysis

For the quantitative study, a pre-tested coded questionnaire developed from empirically validated previously used existing tools [36–38] was used (S1 File). It was reviewed by a water, sanitation and hygiene expert, and discussed amongst the authors, and revised. An informed consent document (S2 File) was used to get consent from prospective participants before data collection. The lack of a more uniform method of measuring and reporting latrine use was reported [39]. To predict factors influencing latrine use (outcome variable), participants were asked how they frequently used their latrines over the previous week [7, 40] using responses ‘Always/Usually used’, ‘Never used’ and ‘Sometimes used’. Measurement was based on 5-day week latrine recall with ‘Always/Usually used latrine’ (at least once every day, ≥ 5 events), ‘Sometimes used the latrine’ (no use in some of the 5 days, but ≠ 0) and ‘Never used the latrine’ (no use in all the 5 days, 0 events). The ‘Always/Usually used latrine’ category was assumed sustained use. Further, respondents identified the main drivers and barriers to latrine use. Adapting the BVIP latrine to climate change (outcome variable) was investigated by asking participants whether they intended to use any adaptation strategy for their latrines using responses ‘Yes’ or ‘No’. Predictor variables were considered from the questionnaire for latrine use and adaptation to climate change (demography and latrine-based). The ‘Yes’ category was assumed that a household would have the intention to adapt its latrine to climate change. The questionnaire items on local climate change adaptation strategies were derived from literature [41, 42] and the authors’ personal experiences working with rural communities in water, sanitation and hygiene interventions.

A latrine inspection checklist (S3 File) on the construction and use of the BVIP latrine was used to determine completeness and correctness of its construction on site. A focus group discussion (FGD) was held in a randomly selected village which did not participate in the quantitative study for each of the five wards. The sixth was held in a ward and village selected by simple random means by two field supervisors. A focus group guide was used (S4 File) following a modified (with written permission) FGD technique framework (S1 Fig) by Nyumba et al. [43]. Participants discussed perceived drivers and barriers for sustained use of the BVIP latrine, and how they adapt it to climate change based on attitudes, motivations, individual experiences or opinions. A moderator and an assistant facilitated the audio-recorded FGD.

Data from completed questionnaires were entered into SPSS version 21.0 [44], cleaned by double entry and finally by cross checking randomly selected 10% of the completed questionnaires and checklists before being imported into STATA version 16 [45] for analysis. Multinomial logistic regression was used to determine predictor variables for latrine use (the dependent variable had three categories). Binary logistic regression was used to determine predictor variables for intending to adapt a household BVIP latrine to climate change (response variable with two categories). Deductive thematic analysis was used to analyse qualitative data (semantic themes) according to the framework by Braun and Clarke [46] (S5 File). Audio-recorded FGDs were transcribed verbatim, coded, similar codes clustered together into several categories, and themes were generated by organising categories underpinned by a central concept. Analyses were done in NVivo 12 [47] and imported into MS Word. Coding was done by two independent investigators, discussed and reached consensus with a third. A set of preliminary codes were developed a priori from literature regarding the use of latrines by households and how they behave or act to the effects of climate change on their latrines in rural communities of LMICs. The codes were applied to transcribed text, reviewed, renamed and merged with others to better capture the data. Others were dropped from the final list of codes used for analysis.

### Ethical approval

The study protocol was approved by an institutional ethics review board (662/2019) and a health ministry at provincial and district levels. All participants provided their informed consent in writing. Participation was voluntary and no compensation was paid.

## Results

### Demographic characteristics of respondents in the cross-sectional survey

Households with BVIP latrines were 238 (30.1%). Table 1 shows that respondents from households owning BVIP latrines were mainly female (73.9%), married (89.5%), belonged to the 36–45 years of age group (30,7%), and were of the *korekore* (60.9%) and *Chikunda* (25.6%) ethnic origins. “Other’ under ethnicity indicates nine small ethnic groups.

**Table 1:** Demographic characteristics of respondents at households with BVIP latrines, Mbire district, northern Zimbabwe, 2021 (*n* = 238).

Variable	Categories	Frequency	%
1. Sex	Male	62	26.1
	Female	176	73.9
2. Marital status	Married	213	89.5
	Single	25	10.5
3. Age group /years	18–25	43	18.1
	26–35	47	19.7
	36–45	73	30.7
	46–55	46	19.3
	> 55	29	12.2
4. Educational level	No formal education	28	11.8
	Primary	142	59.7
	Secondary	58	24.4
	Tertiary	10	4.2
5. Ethnicity	*Korekore*	145	60.9
	*Chikunda*	61	25.6
	Foreign	1	0.4
	Other	31	13.1
6. Religion	Christianity	197	82.8
	Traditional	23	9.7
	Muslim	6	2.5
	None	12	5.0
7. Approximate monthly household income /USD	Less than 50	159	66.8
	50–100	42	17.6
	101–200	26	10.9
	Greater than 200	11	4.6
8. Household size	≤ 2	11	4.6
	3–5	115	48.3
	> than 5	112	47.1
9. Number of cattle owned by household	None	174	73.1
	≤ 3	22	9.2
	4–5	28	11.8
	> 5	14	5.9
10. Residency period /years	< 1	11	4.6
	2–10	68	28.6
	11–20	57	23.9
	> 20	102	42.9
11. Nature of household	Nucleus	132	55.5
	Extended	106	44.5

### Characteristics of inspected BVIP latrines at households

**Fig 1:**
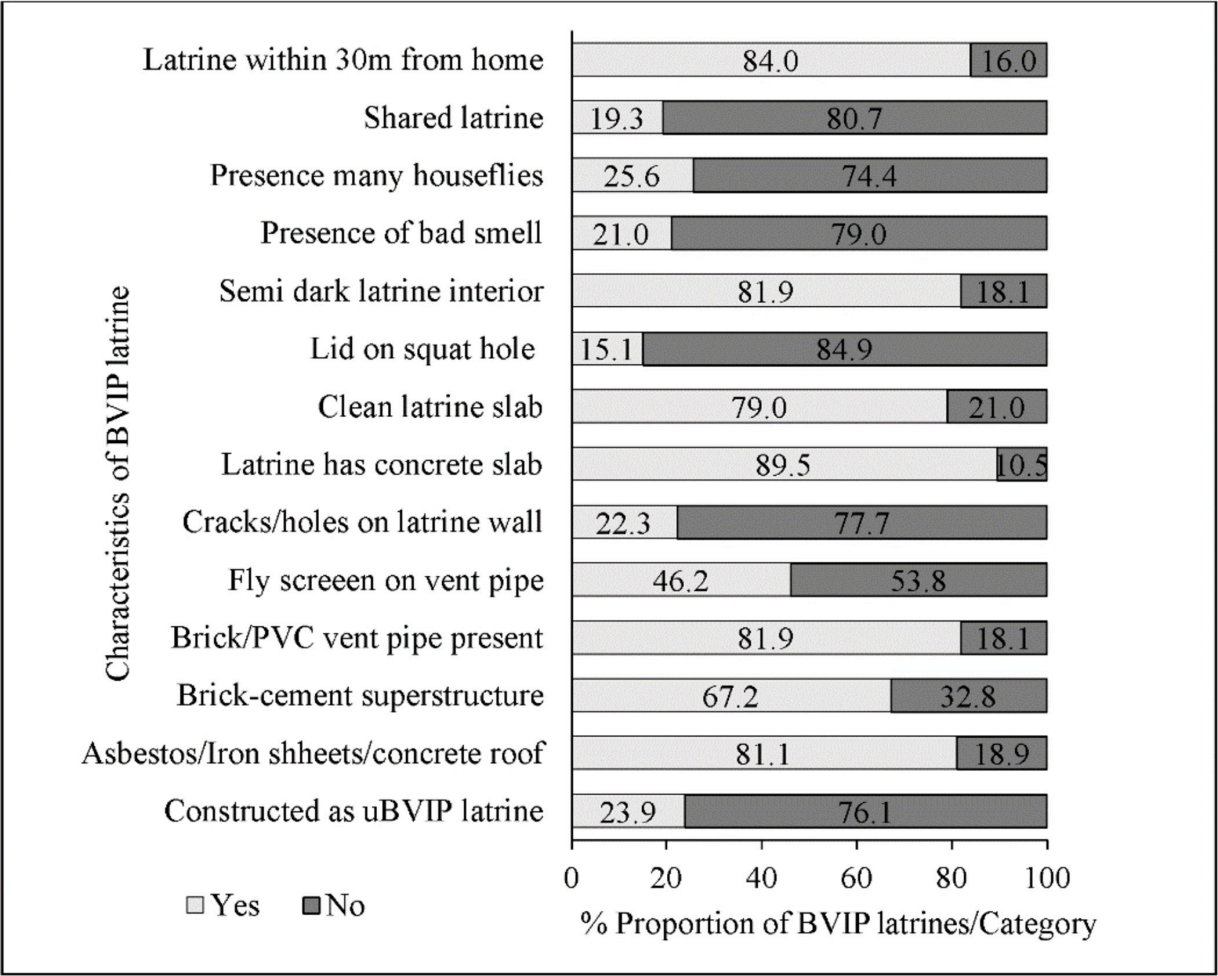
Characteristics of household BVIP latrines in rural villages of Mbire district, northern Zimbabwe, 2021 (n = 238).

**Fig 2:**
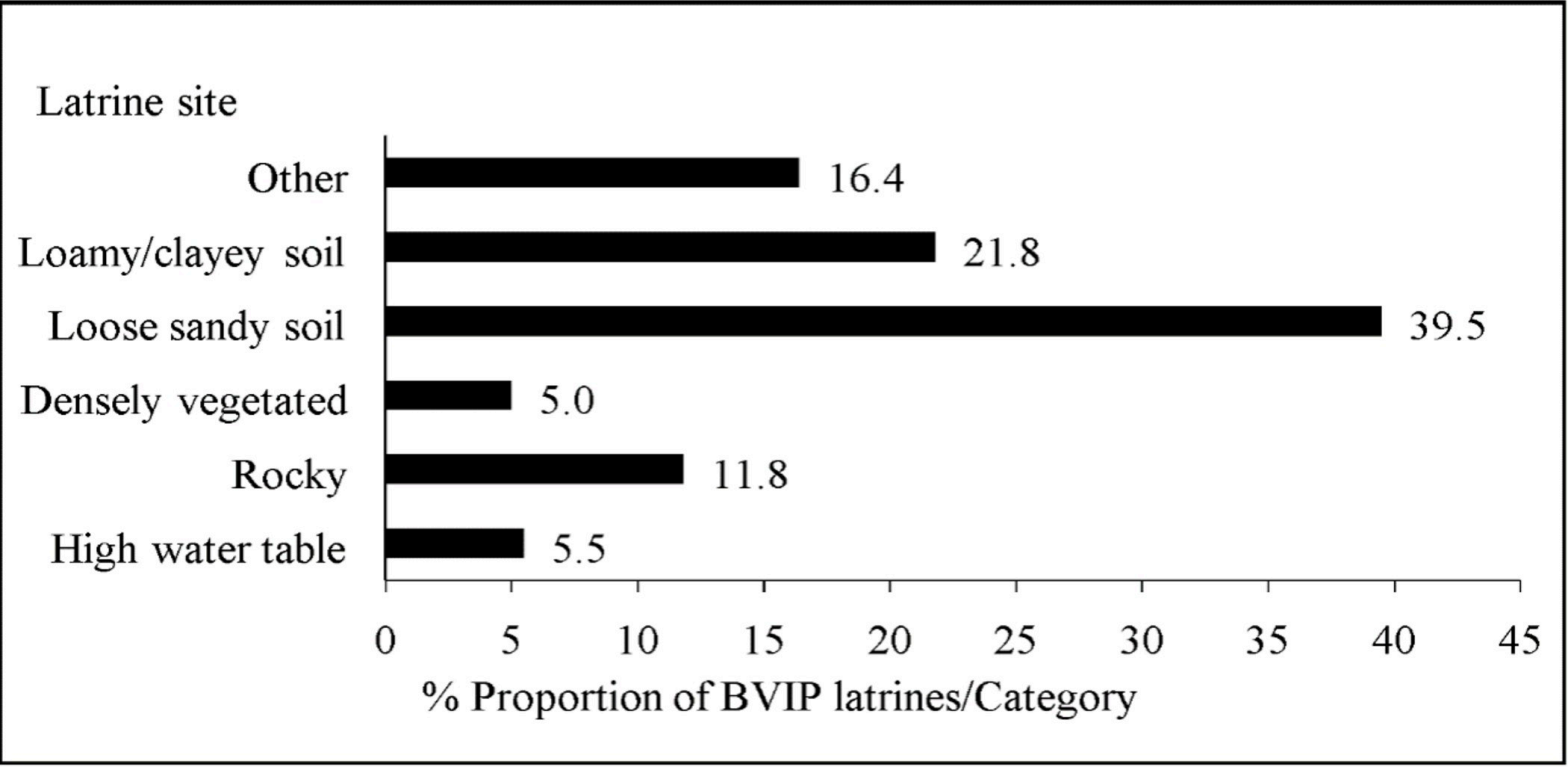
Description of household BVIP latrine sites in Mbire district, northern Zimbabwe, 2021 (n = 238).

A completed BVIP latrine which was constructed in stages while in use was considered an upgradable BVIP latrine version in this case, otherwise it was generally considered a BVIP latrine in the discussion. Most BVIP latrines (67.2%) had superstructures made of fired farm bricks and cement, and 89.5% of them had concrete slabs (Fig 1). Some latrines had no vent pipes (18.1%) or fly screens (53.8%). Squat holes on the slabs had lids in some latrines (15.1%). Thirty-eight latrines (16.0%) were located more than 30 m away from the home. About 40% of the latrines were constructed on sandy soil (Fig 2).

### Latrine use patterns

**Fig 3:**
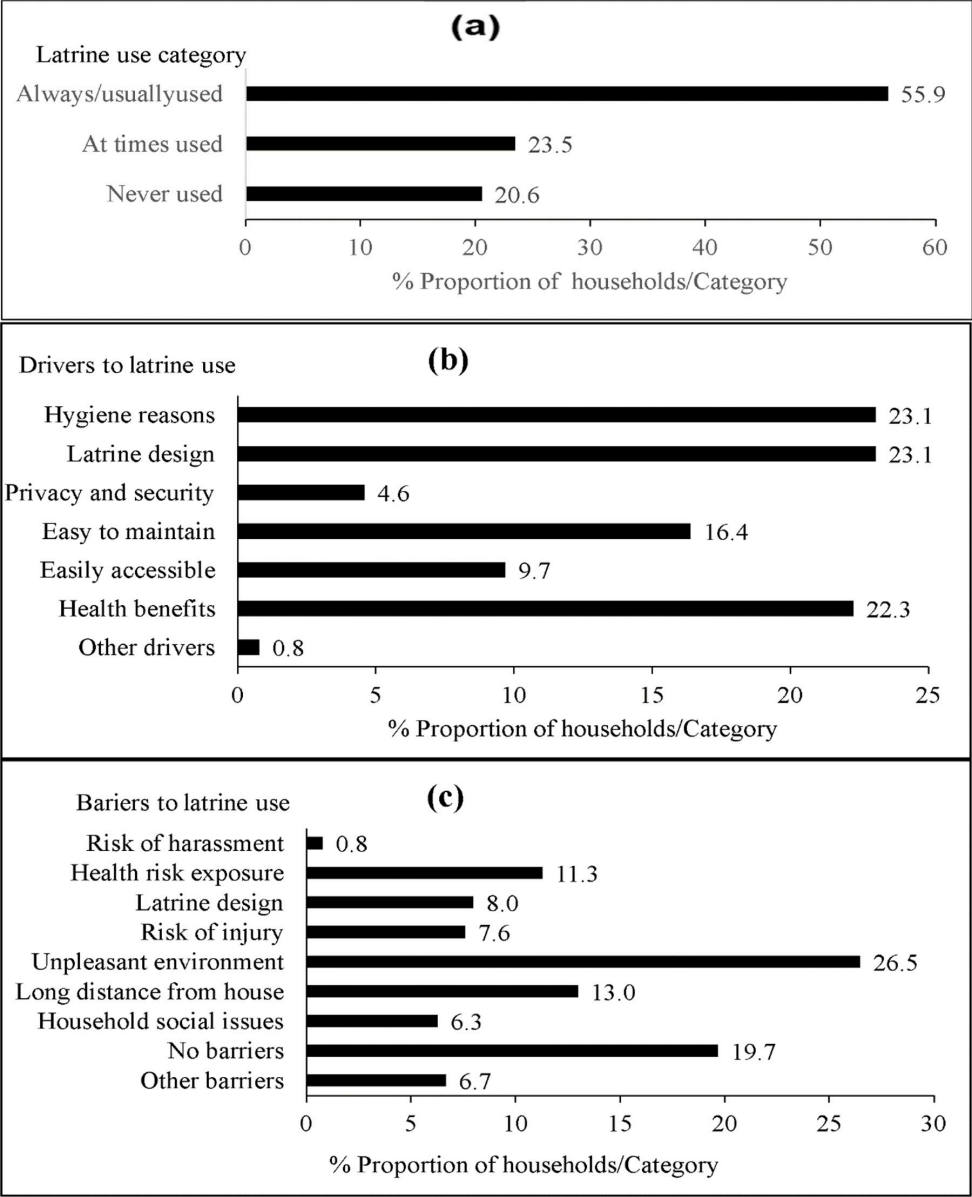
Latrine use in the previous week (a), drivers (b), and barriers (c) to sustained latrine use among households owning BVIP latrines in Mbire district, northern Zimbabwe, 2021.

There was moderate self-reported use of BVIP latrines (55.9%) by house heads in the previous week while 20.6% of them never did (Fig 3A). Self-reported drivers to sustained latrine use were completed superstructure and absence of cracks/holes on the latrine, that is, its design (23.1%), hygienic environment (23.1%), perceived health benefits (22.3%) and easy to maintain (16.4%) (Fig 3B). About 27% of the participants indicated that an unclean latrine environment was a major barrier to its use. Other households (19.7%) did not report any barriers to use their latrines (Fig 3C).

#### Disposal of children’s stools

**Fig 4:**
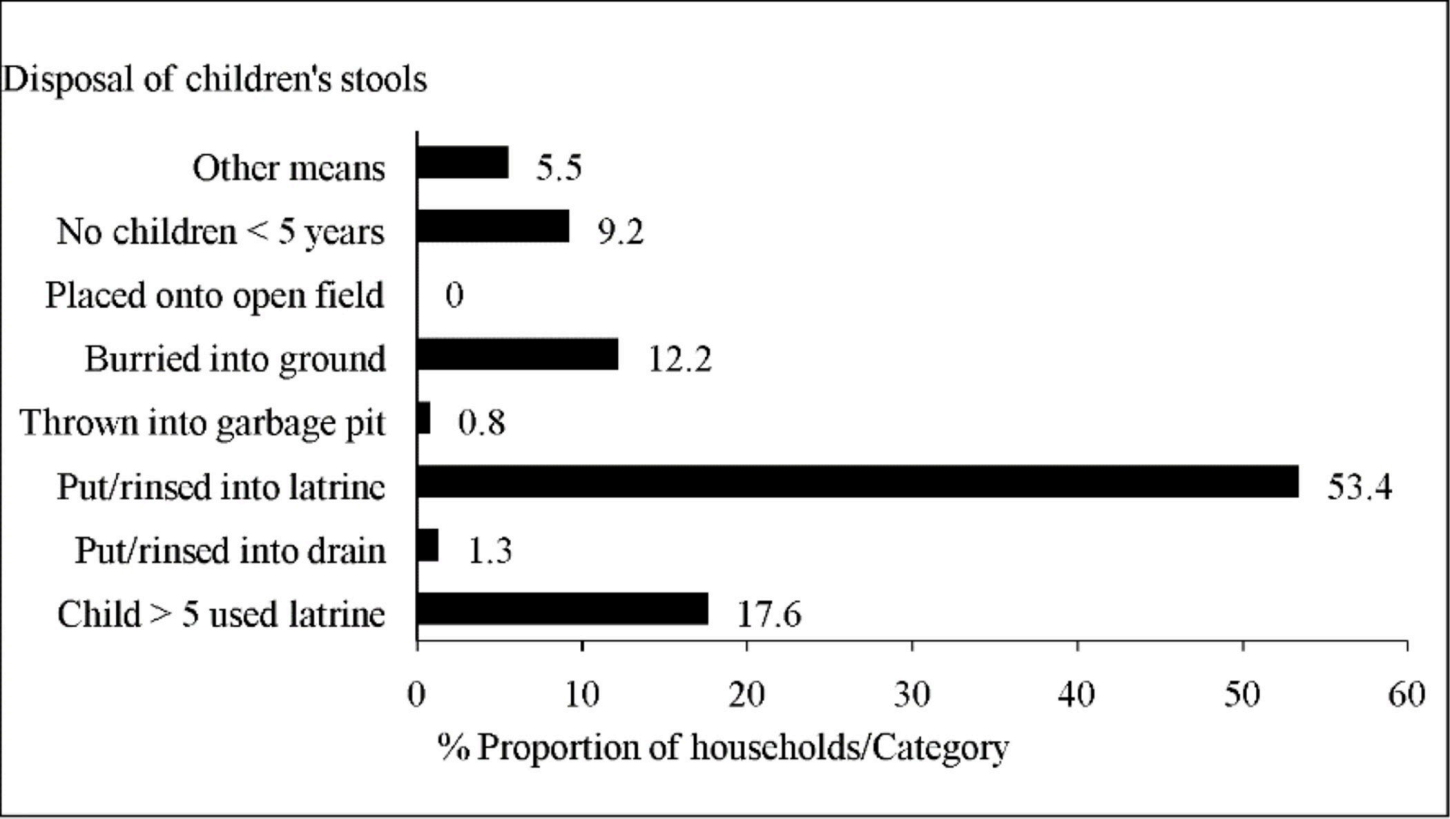
Safe disposal of children’s stools using the BVIP latrine in Mbire district, northern Zimbabwe, 2021 (n = 238).

More than half of the respondents (53.4%) indicated that they dispose of children’s stools into the BVIP latrine (Fig 4). Further, children greater than five years of age were reported to use the latrine (17.6%). A few households (4.2%) reported to use unsafe methods to dispose of children’s stools.

### Determinants of latrine use

Four individual- and seven latrine-based variables used in the multinomial logistic regression model (main effects) were not significantly associated (p > 0.05) with latrine use (Table 2). Seven variables which were significant (p < 0.05) were used in the *post hoc* analysis (Table 3). Statistically significant (p < 0.05) variables have p values in bold. Two individual-level contextual predictors (age and religion of house head) of latrine use were determined. *Post hoc* results show a significant increased likelihood of reporting *Always/Usually used* the latrine versus *Never used* it for the 26–35 years of age group than the reference category of > 55 years of age group (OR = 13.46, 95% CI = 2.01, 89.79, p = 0.007). The 36–45 years of age group was significantly more likely to report *Always/Usually used* the latrine versus *Sometimes used* it than the > 55 years of age group (OR = 4.08, 95% CI = 1.07, 15.60, p = 0.04). A house head of traditional religion was significantly more likely than one of none to report *Sometimes used* the latrine versus *Never used* it (OR = 25.28, 95% CI = 0.95, 66.91, p = 0.046).

**Table 2:** Main effects of latrine use.

Predictor variable	Likelihood ratio	Chi-Square	df	p-value
Intercept	209.90			
Clean latrine slab without faeces	213.02	3.13	2	0.209
Few houseflies observed around latrine	212.68	2.78	2	0.249
Latrine inside is dark	212.18	2.28	2	0.320
Open defaecation	210.63	0.73	2	0.695
Build standard BVIP latrine	210.80	0.90	2	0.637
Add wood ash pit	212.05	2.15	2	0.342
latrine is less than 30 m from the home	212.04	2.14	2	0.343
Sex	228.88	4.63	2	0.099
Marital status	227.54	3.28	6	0.773
Age group	229.08	4.83	8	0.776
Education level	229.17	4.92	6	0.554

**Table 3:** Parameter estimates of the multinomial logit latrine use model showing the effect of individual, household and technology-level predictors on sustained use a BVIP latrine rural households in Mbire district, northern Zimbabwe (n = 238).

		Always/Usually Vs. Never			Sometimes Vs. Never			Always/Usually Vs. Sometimes		
Variable (Reference category)	Categories	Odds Ratio	95% CI	p value	Odds ratio	95% CI	p value	Odds ratio	95% CI	p value
Age group / years (> 55)	18–25	1.34	0.23, 7.69	0.75	0.55	0.08, 3.59	0.53	2.45	0.51, 11.9	0.27
(Nearest 1)	26–35	13.46	2.01, 89.79	**0.007**	4.75	0.64, 35.20	0.13	2.84	0.67, 11.96	0.16
	36–45	2.38	0.56, 10.20	0.24	0.58	0.12, 2.94	0.51	4.08	1.07, 15.6	**0.04**
	46–55	2.31	0.47, 11.29	0.30	0.96	0.17, 5.37	0.96	2.41	0.59, 9.82	0.22
Household monthly income /USD (> 200)	≤ 50	0.22	0.02, 2.91	0.25	1.60	0.06, 41.65	0.78	0.14	0.01, 1.52	0.11
	51–100	0.72	0.04, 12.43	0.82	8.83	0.26, 29.57	0.22	0.08	0.01, 1.07	**0.047**
	101–200	0.06	0.003, 0.98	**0.047**	0.15	0.004, 5.88	0.31	0.38	0.02, 6.38	0.50
Religion (None)	Christianity	2.30	0.32, 16.61	0.41	13.02	0.81, 20.97	0.07	0.18	0.02, 2.0	0.16
	Traditional	7.59	0.58, 98.87	0.12	25.28	0.95, 66.91	**0.046**	0.30	0.02, 4.09	0.37
	Muslim	0.13	0.01, 2.6	0.18	0.57	0.01, 24.48	0.77	0.23	0.01, 8.32	0.42
Household size (> 5)	≤ 2	24.99	1.27, 49.26	**0.03**	7.27	0.20, 26.27	0.28	3.44	0.33, 35.73	0.30
	3–5	1.70	0.65, 4.42	0.28	1.30	0.45, 3.74	0.63	1.31	0.59, 2.92	0.51
No. of cattle owned (> 5)	None	7.02	0.90, 54.55	0.06	1.35	0.19, 9.72	0.76	5.19	1.08, 24.94	**0.04**
	≤ 3	50.88	2.09, 124.1	**0.02**	31.0	1.25, 76.66	**0.036**	1.64	0.28, 9.77	0.59
	4–5	4.98	0.52, 47.76	0.16	2.36	0.25, 21.93	0.45	2.11	0.35, 12.69	0.41
Residence period /years (> 20)	< 2	0.16	0.02, 1.58	0.12	0.06	0.003, 1.42	0.08	2.63	0.19, 36.39	0.47
(Nearest 1)	2–10	0.40	0.12, 1.29	0.12	1.04	0.29, 3.75	0.96	0.38	0.14, 1.03	0.06
	11–20	0.25	0.08, 0.79	**0.02**	0.25	0.07, 0.93	**0.039**	1.02	0.38, 2.77	0.97
No odour from Latrine (No)	Yes	0.49	0.22, 1.08	0.078	1.21	0.52, 2.83	0.67	2.46	1.17, 5.17	**0.017**

Four household-level contextual predictors (household size, residence period, income and cattle ownership) of latrine use were determined. The highest household income of > 200 USD was the reference category. A house head from a household with monthly income from all sources falling within the 51–100 USD category was significantly less likely than one from the reference to report *Always/Usually used* latrine versus *Never used* it (OR = 0.08, 95% CI = 0.01, 1.07, p = 0.047), similarly for one from the 101–200 USD category (OR = 0.06, 95% CI = 0.003, 0.98, p = 0.047). Results show that the smallest household was significantly more likely than the largest (reference) to report Always/Usually used the latrine versus Never used it (OR = 24.99, 95% CI = 1.27, 49.26, p = 0.03).

Having no cattle at the household was found to be both significantly more likely than having more than five to have *Always/Usually used* the latrine versus *Sometimes used* it (OR = 5.19, 95% CI = 1.08, 24.94, p = 0.04), and *Sometimes used* the latrine versus *Never used* it (OR = 31.00, 95% CI = 1.25, 76.66, p = 0.036). There was significantly increased likelihood of a household with ≤ 3 cattle than with > 5 to report *Always/Usually used* the latrine versus *Never used* it (OR = 50.88, 95% CI = 2.09, 124.1, p = 0.020). A household residence period of 11–20 years in the ward than > 20 years had a 75% decrease in the likelihood of reporting *Always/Usually used* the latrine versus *Never used* it (OR = 0.25, 95% CI = 0.08, 0.79, p = 0.020), and *Sometimes used* it versus *Never used* the latrine (OR = 0.25, 95% CI = 0.07, 0.93, p = 0.039).

A single technology-based predictor (odour) was determined. Perceiving the BVIP latrine as having no obnoxious (bad) smell was significantly 2.46 times more likely than having it, to report *Always/Usually used* the latrine versus *Sometimes used* it (OR = 2.46, p = 0.017, 95%CI = 1.17, 5.17).

### Predictors of adapting a household BVIP latrine to climate change

Two individual-level variables (sex and age group) were significant in predicting the intention to adapt a latrine to climate change (Table 4). Category in brackets () after the predictor variable denote reference category. The Hosmer and Lemeshow test gave a Chi-square value of 6.209, df = 8, p = 0.624. The model specificity was 54.3% while its sensitivity was 82.9%. Overall classification was 71.8%. P values in bold denote statistically significant (p < 0.05). Female house heads were 2.293 times significantly more likely than their male counterparts to express an intention to adapt household BVIP latrines to climate change (OR = 2.293, p = 0.038, 95% CI = 1.046, 5.027). Older house heads, 36–45 years of age group had significantly greater likelihood than the 18–25 years of age group to indicate the intention to adapt their latrines to climate change (OR = 4.477, p = 0.007, 95% CI = 1.516, 13.204), so was the 46–55 years of age group than the reference category (OR = 4.445, p = 0.012, 95% CI = 1.406, 15.483). Although the oldest group (> 55 years of age group) had greater likelihood than the 18–25 years of age group of intending to adapt, it was not statistically significant (OR = 2.444, p = 0.207, 95% CI = 0.609, 9.809).

**Table 4:** Binomial logistic regression model showing the effect of individual, household and technology-level predictors on the intention to adapt a BVIP latrine to climate change for rural households in Mbire district, northern Zimbabwe (n = 238).

Predictor variable (Reference category)	Categories	B	Wald statistic	p value	Odds Ratio	95% CI
Sex (Male)	Female	0.830	4.294	**0.038**	2.293	1.046, 5.027
Marital status (married)	Single	0.473	0.696	0.404	1.605	0.528, 4.880
Age group /years (nearest 1) (18–25)	26–35	0.328	0.388	0.533	1.388	0.495, 3.898
	36–45	1.498	7.362	**0.007**	4.477	1.516, 13.204
	46–55	1.540	8.332	**0.012**	4.665	1.406, 15.483
	> 55	0.894	1.589	0.207	2.444	0.609, 9.809
Household income in USD (< 50)	51–100	1.566	9.582	**0.002**	4.790	1.775, 12.927
	101–200	0.046	0.008	0.929	1.047	0.379, 2.889
	> 200	0.665	0.652	0.419	1.945	0.387, 9.779
Household size (≤ 2 members)	3–5	1.644	4.263	**0.039**	5.177	1.087, 24.655
	> 5	1.832	4.780	**0.029**	6.247	1.209, 32.282
Number of cattle owned (none)	≤ 2	- 0.671	1.550	0.213	0.511	0.178, 1.470
	3–5	- 1.206	5.338	**0.021**	0.299	0.108, 10,833
	> 5	0.600	0.668	0.414	1.822	0.432, 7.684
Open defaecation (No)	Yes	0.279	0.701	0.402	1.322	0.688, 2.543
Built raised latrine (No)	Yes	0.063	0.033	0.856	1.065	0.538, 2.111
Built conventional BVIP (No)	Yes	0.079	0.056	0.812	1.082	0.565, 2.073
Add wood ash into the pit (No)	Yes	0.212	0.354	0.552	1.236	0.616, 2.479
Bath in the latrine (No)	Yes	0.881	6.580	**0.010**	2.414	1.231, 4.733
Latrine built on raised ground (No)	Yes	- 0.993	9.039	**0.003**	0.370	0.194, 0.708
Constructed emergency latrines (No)	Yes	- 0.056	0.028	0.968	0.946	0.489, 1.829

Household-level predictors included households with a monthly income of 51–100 USD which were significantly 4.79 times more likely than those with less than 50 USD to demonstrate the intention to adapt their latrines to climate change (OR = 4.790, p = 0.002, 95% CI = 1.775, 12.927). Increased likelihood of the intention to adapt the BVIP latrine was evident on larger household sizes than smaller ones, for 3–5 than ≤ 2 members (OR = 5.177, p = 0.039, 95% CI = 1.087, 24.655) and > 5 members than the reference category (OR = 6.247, p = 0.029, 95% CI = 1.209, 32.282). A decreased likelihood of households with 3–5 cattle than those with none was observed for the intention to adapt latrines to climate change (OR = 0.299, p = 0.021, 95% CI = 0.018, 10.833). A technology-level predictor established was bathing in the latrine (also behaviour-level predictor). Households bathing in the latrine were significantly more likely to indicate intention to adapt it to climate change than those which did not (OR = 2.414, p = 0.010, 95% CI = 1.231, 4.733). Further, there was evidence of significantly decreased likelihood of households with latrines built on raised ground than those without to adapt it to climate change (OR = 0.370, p = 0.003, 95% CI = 0.194, 0.708).

### Reasons for not adapting the BVIP Latrine to climate change

**Fig 5:**
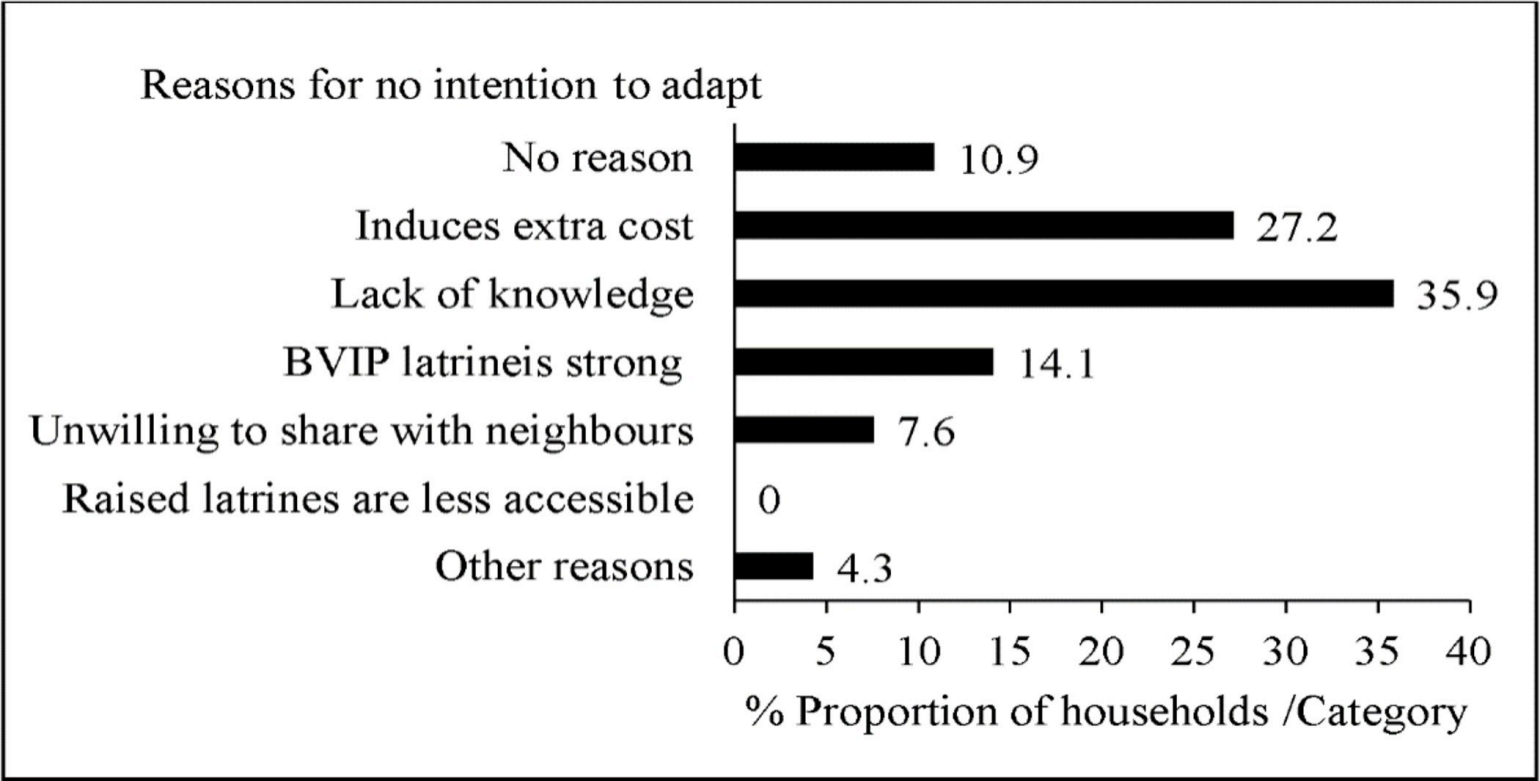
Reasons for no intention to adapt household BVIP latrines to climate changes in Mbire district, northern Zimbabwe, 2021 (n = 92).

About 38.7% of households with BVIP latrines indicated that they had no intention of adapting their latrines to climate change. Fig 5 shows that most reasons that were given by house heads were lack of knowledge of latrine adaptation to climate change (35.9%), perceived high cost associated with adaptation strategies (27.2%) and others viewed the BVIP latrine as a strong design that does not need adaptation to climate change (14.1%).

### Adaptation strategies of BVIP latrine to climate change among households

**Fig 6:**
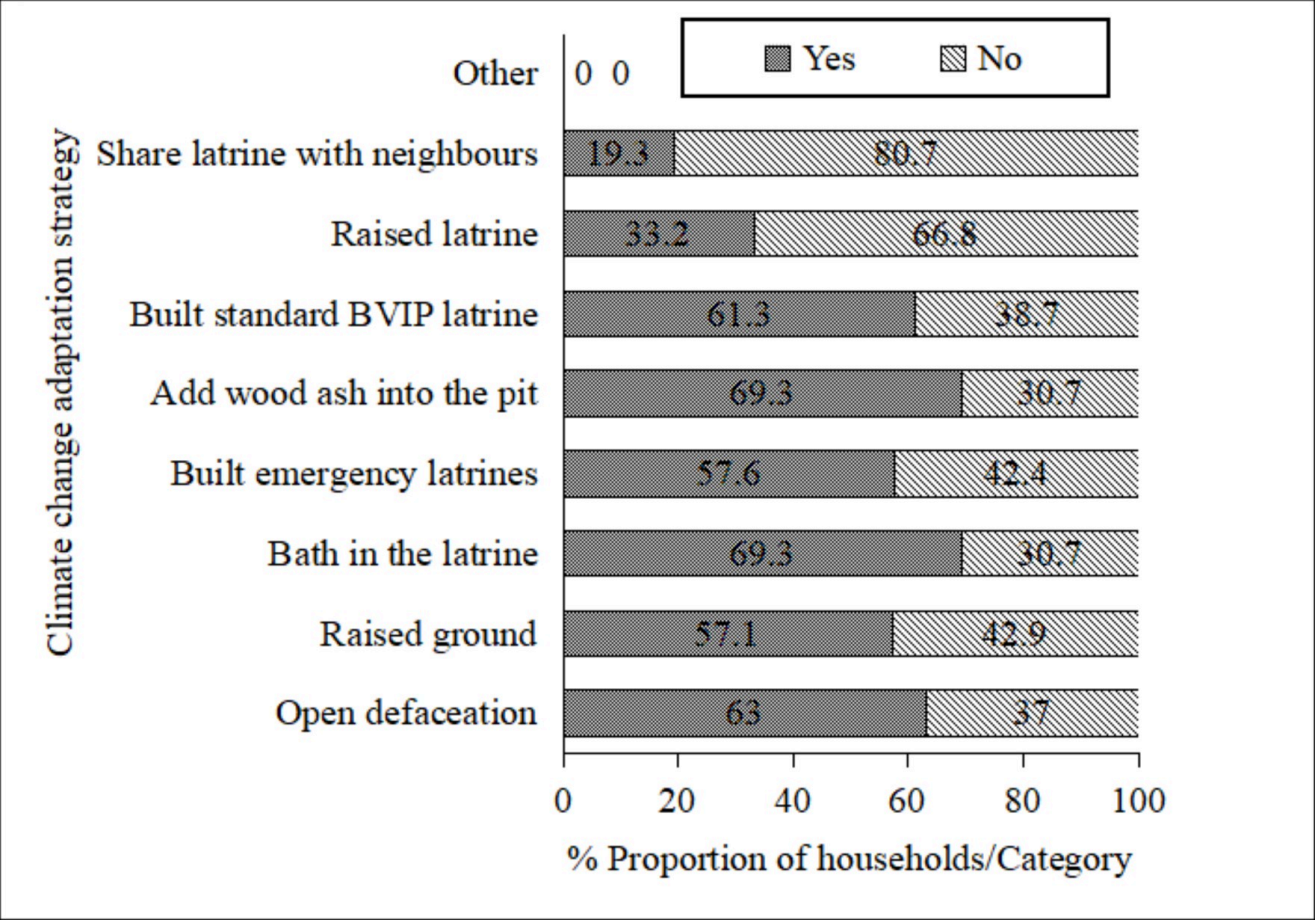
Reported adaptation strategies (survey) of household BVIP latrines to climate change in Mbire district, northern Zimbabwe, 2021 (n = 238).

Addition of wood ash into the latrine pit and bathing in the latrine (69.3%) to control bad odour emerged the commonest climate change strategies (Fig 6). Most respondents indicated that the standard BVIP latrine design was resilient to climate change effects (61.3%). Due to additional cost associated with improving the latrine design, some households indicated that they would opt for open defaecation (63.0%). Sharing of latrines with neighbours was the least common climate change adaptation strategy of the BVIP latrine (19.3%).

### Characteristics of participants and focus groups in the qualitative study

Table 5 indicates that 39 house heads (72.2%) participated in focus groups (ave. 7 participants). About half of the participants (51.3%) were female. Audio-recorded FGDs were held within 85 minutes (68–83, ave. 75.5 minutes). FDG denoted 9* throughout this report denotes it was done by field supervisors.

**Table 5:** Characteristics of participants in focus groups, Mbire district, northern Zimbabwe, 2021 (n = 39).

	Ward where a focus group discussion was held							
Characteristic	1	5	9	10	15	9*	Total	Average
Venue	School	School	Clinic	Clinic	Clinic	Clinic	-	-
Duration (minutes)	68	71	83	81	76	74	437	75.5
Number of participants	6	9	7	6	6	5	39	7
Females	3	3	5	2	4	3	20	4
Highest frequency age group	> 45	26–35	36–45	36–45	> 45	36–45	-	-
Post-primary education	3	5	3	5	3	2	21	4
Community leader	0	1	0	1	0	0	2	-
Professionally employed	2	0	2	2	0	0	6	1

### Sustained BVIP latrine use: Evidence from focus groups

**Fig 7:**
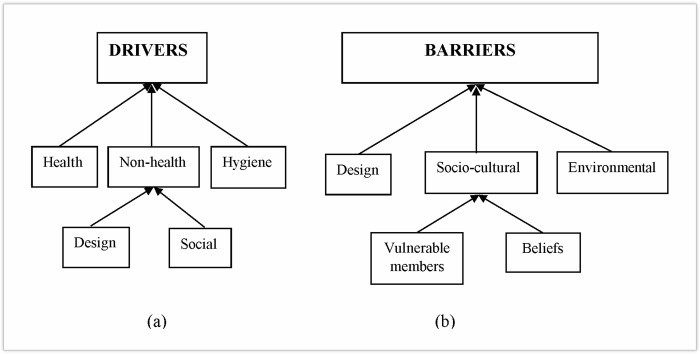
Focus group perceived drivers and barriers to users of the BVIP latrine, Mbire district, northern Zimbabwe, 2021.

Three main perceived multilevel drivers (health, non-health and hygiene) and three barriers (design, environmental and socio-cultural) with sub-factors in some instances, were determined from FGDs (Fig 7) and summarised in Table 6.

**Table 6:** Summary of perceived drivers and barriers (multi-level) to users of BVIP latrine from focus groups, Mbire district, northern Zimbabwe 2021.

Perceived drivers	Perceived barriers
Offers dignity, privacy and security	Not always used when distant from the home
Prevents contracting diarrhoeal diseases to the family	Instils fear of collapse if latrine has observable cracks
Containment of faecal matter gives a clean home Environment	Semi-dark interior scares users for fear of snakes, bats and mosquitoes
Prevents contamination of food and water with faecal pathogens	May not be suitable for extended families with in-laws (especially one unit)
Controls odour and houseflies	Faecal matter on slab prevents use
Long life and strong when well built	Without odour and fly control cannot be used
Flexible to be built over time (upgradable) using local resources	May not be accessible to the elderly, very young and physically handicapped
Can alternatively be used as a bathroom	
Concrete slab is easily cleaned	
Safe disposal of children’s faeces	

### Drivers to latrine use

#### Health, non-health and hygiene drivers

Results from the FGDs indicated that sustained latrine use seem to be driven by perceived health, non-health and hygiene benefits. The non-health driver was subdivided into two; latrine design and social considerations (Fig 7A). All the three factors were summed in a statement by a female participant (greater than 45 years of age) from ward 1 as follows: “*The BVIP latrine prevents diarrhoeal diseases*, *offers privacy*, *security*, *dignity*, *and is easy to clean*”. Another participant added the potential of latrine for fly control and excreta containment which provide a hygienic environment to motivate the user: “*The BVIP Latrine kills houseflies and prevents them from getting into house*, *provides a hygienic environment*. *It allows disposal of children’s faeces*” (Ward 5, Female, 26–35 years age group).

### Barriers to latrine use

Three main barriers were identified for sustained latrine use. The Socio-cultural barrier was subdivided into two (Fig 7B)

#### Environmental barrier

The use of a BVIP latrine is faced environmental challenges. Pits were reported to fill up with water especially during the rainy season. A participants had this to say: “*The pits are filled with water in the rainy season allowing faecal matter to float near the surface of the pit or overflow*. *This result in family members not using the latrine*. *Also*, *houseflies can move in and out of the pit freely*. *This allows diarrhoeal outbreaks*” (Ward 9, Female, > 45 years age group).

#### Latrine design

Despite owning a BVIP latrine, participants expressed that its design presents barriers to access it, instils fear and has security threats. Participants gave examples of the latrine design barrier: “*The elderly*, *children and physically challenged may fail to access the BVIP latrine*” (Ward 1, Male, > 45 years age group). “*The dark interior of a BVIP latrine is scary to users*, *especially during the night*. *There are reports of having snakes and bats being harboured in the latrine*. *Further*, *in malarial areas*, *the BVIP latrine harbours mosquitoes”* (Ward 15, Female, 18–25 years age group). Use of a latrine was reported limited when it is located further away from the home and not accessible by all including vulnerable members of the household. A male participant (aged > 45 years) from ward 1 noted: “*My BVIP latrine is built some distance away from the house as we could not find an appropriate site near the house*. *I have observed that at night not all of us use it for fear of darkness*. *This also happens when it is raining*” (Ward 1, Male, > 45 years age group).

#### Socio-cultural barrier

Staying with in-laws as an extended family was observed as a barrier to latrine use in two focus group discussions. A participant explained: “*The latrine may not be suitable for an extended family where in-laws are staying together*. *Although very few households still practise this culture*, *health education is removing such taboos*” (Ward 1, Female, 26–35 years age group).

#### Adaptation of the BVIP latrine to climate change from focus groups

**Fig 8:**
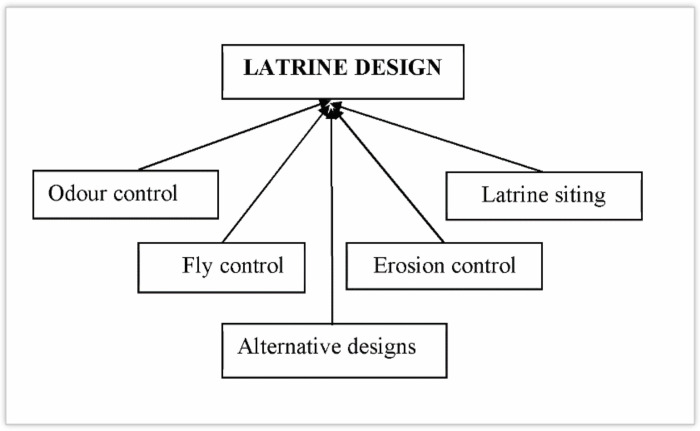
Adaptation strategies of the BVIP latrine to climate change from focus groups, Mbire district, Zimbabwe, 2021.

Results from focus groups suggest that most BVIP latrines were not of the ‘standard 5–7 bag cement model’ expected to operate well and easily cleaned. Fig 8 shows the main thematic areas and Table 7 the adaptation strategies of how rural households adapt BVIP latrine to climate change.

**Table 7:** Adaptation strategies of BVIP latrine to climate change, Mbire district, northern Zimbabwe (Focus groups), 2021.

Adaptation strategy	Approaches by households
Latrine design	Raised slab level
Latrine design	Site latrine on raised ground
Latrine design	Construct standard 5–7 bag cement latrine
Latrine design	Site latrine on firm soil
Latrine design	Construct concrete latrine roof and brick latrine vent pipe
Latrine design	Construct superstructure with fired brick and cement
Odour control	Addition of wood ash in latrine pit
Odour control	Bathing in the latrine adding water into the pit
Erosion control	Build a concrete pavement around the latrine
Erosion control	Construct a contour around the latrine
Insect control	Spraying chemicals onto latrine walls to kill houseflies and mosquitoes
Alternative options	Temporary pit latrines, cat sanitation and the bush/field
Alternative options	Share latrine with neighbours

### Latrine design

#### Construction of the conventional design

The BVIP latrine has ‘a conventional 5–7 bag cement’ design and several upgradable models. Adaptation strategies to climate change are central to its design: “*We used to build only the latrine pit with cement*, *the outer wall with dagga then plaster with cement*. *With the changing climate*, *we are reverting to the standard 5-bag cement BVIP latrine which uses cement throughout*. *This gives a strong structure to withstand rainfall and strong winds*” (Ward 10, Male, 36–45 years age group). Adequate cement is needed to prevent latrines from collapsing. The standard 5–7 bag cement is considered strong. However, if households build modified design with less cement it is subject to collapsing. Collapsing of latrines was mentioned five times across FGDs. A participant noted: “*When built on sandy soil without adequate cement and reinforcement*, *BVIP Latrines collapse in the rainy season*” (Ward 1, Male, 36–45 years age group).

#### Odour and housefly control

Participants mentioned adding wood ash and bathing in the latrine and to control odour and houseflies. The addition of wood ash into the latrine pit appears to be common practice mentioned eight times across FGDs. They explained: “*In summer where temperatures are very high*, *we bath in the latrine to reduce strong odours by reducing temperature*. *Alternatively*, *we add wood ash into the pit*” (Ward 10, Male, 36–45 years age group). Further, spraying chemicals was also mentioned: “*In hot weather and the rainy season we may experience large numbers of houseflies which can move in and out of the pit easily*. *So we spray chemicals into the pit and latrine interior to kill them*” (Ward 9*, Female, 36–45 years age group).

#### Erosion control

Participants explained that trenching and paving the ground around the latrine with concrete were two ways of controlling erosion: “C*onstruct a shallow diversion trench around the latrine in the rainy season so that water flows away without filling the pit*”, (Ward 9, Female, > 45 years age group), and “*I have seen some households using extra cement to pave the surrounding of the latrine with concrete to avoid soil erosion which leads to collapse of the latrine*”. (Ward 1, Male, 36–45 years age group).

#### Latrine siting

The BVIP latrine should be constructed on firm soil and raised areas to prevent it from collapsing and the pit filling up with water. Two participants had this to say: “*To make latrines accessible in times if heavy rainfall*, *during construction*, *raised latrines can be used*, *or construct latrines on raised areas*” (Ward 10, Male, 36–45 years age group). Another participant added: “W*e construct raised BVIP latrines in places where the pit cannot be deep enough (rocky) or low-lying places which can allow runoff to accumulate*” (Ward 15, Male, > 45 years age group).

#### Alternative sanitation options

Participants identified ‘cat sanitation’ and the bush (open defaecation) as alternative sanitation options to using the BVIP latrine when made inaccessible by climate change effects. A few households shared sanitation facilities with their neighbours. A participant explained: “*In situations where sharing of latrines is not a viable option*, *household members end up using the bush*, *practising open defaecation*” (Ward 10, Female, 36–45 years age group). In another focus group, a participant indicated: “*In times of high rainfall events or at night*, *the cat sanitation is used instead of the BVIP latrine*” (Ward 1, Female, 36–45 years age group).

## Discussion

The current study presents one of the few, or first report on perceived drivers and barriers of sustained use of a ventilated improved pit (VIP) latrine design (locally called BVIP latrine) for over four decades of technology implementation in rural Zimbabwe. This was because of a long-standing policy which encouraged the implementation of this home-grown innovation without considering appropriate alternative sanitation options to suit different environmental settings, even in the face of climate change. This is envisioned in the new national sanitation policy draft of Zimbabwe [48]. Focus group discussions appear to unearth more latrine use drivers and barriers of a social nature, not found significant in the quantitative study. Results from the quantitative study show that contextual factors at the household level appeared to influence latrine use in the study area.

From the quantitative study it was found that some households did not use their BVIP latrines. This is consistent with previous reports where various latrine designs were not used [6, 15, 49]. Reasons for non-use of latrines varied from technology, socio-cultural to hygienic latrine environment at individual and household levels [30]. Old age and lack of a religion appeared not to favour always using a latrine. The age of all household members, as opposed that of the house head in the current study, were shown to influence latrine use [10, 15]. In rural Ecuador, elderly men were found less likely to use a latrine [50]. This could be explained by attitude or beliefs. Religion was not selected as a predictor variable in most studies evaluating latrine use.

High household income and a long residence period were found to increase the likelihood of latrine use. No cause-effect relationships were established and these predictor variables were rarely used in similar study settings. Current results corroborate with the general observation that latrines with bad odour are not ‘always’ used. Odour from human excreta influences latrine use due to social, moral, aesthetic, and disease-related concerns [51]. Detection of odour form a well-constructed and functional VIP latrine indicates faulty odour control due to poor maintenance. Other studies indicated that socio-cultural factors at the household level were considered the main latrine use predictors [50] while an interplay of the technology, social and contextual factors was attributed to latrine use [52].

Participants in the quantitative study had either no formal (11.8%) or primary (59.7%) education but indicated high latrine use. Results show that education was not a significant predictor of BVIP latrine use. A similar conclusion was arrived at by Sinha [15] in a CLTS evaluation study. However, this finding is contradictory to other findings [16–18, 49]. A possible explanation could be that other than formal education, environmental health technicians (EHTs) stationed at rural health centres in the study area and village health workers staying in the villages freely give awareness and knowledge on the use of a BVIP latrine as part of their routine work. This arrangement augurs well for community support especially where household heads lack knowledge or awareness on sanitation issues.

Results from the qualitative study indicated that a hygienic latrine environment (absence of foul odour, houseflies and faecal matter on the slab) was a driver for sustained latrine use. This is consistent with a wide literature [17, 50, 53]. Possible reasons for an unclean latrine environment could be bad attitude, use by young children or pit floatation with excreta especially in the rainy season. The presence of faeces on the latrine floor was reported to provoke open defaecation [54]. The concrete slab of the BVIP latrine can easily be cleaned to provide a hygienic environment for use.

The dark interior and poor quality of the latrine were mentioned in focus groups as barriers to latrine use. A poorly-built BVIP latrine may compromise its design (strength, life, durability) and operation (odour and fly control) which influences its use. Results from the observation checklist showed that some latrines lacked vent pipes or fly screens, and had odour and many houseflies. If local communities ignored the special design specifications of constructing conventional VIP latrines for odour and fly control, then such latrines may not the best options for the area [55]. Instead, latrine modifications or alternative options may be suggested for sustained use. Despite being mentioned in focus groups, hygienic latrine environment, its design and educational level of the house head were not determinants of latrine use from the quantitative study. Therefore, a mixed methods study appears useful to explore experiences by households which could otherwise not be unearthed by a questionnaire alone.

Sandy soil contributed to latrine pit floatation with faecal matter, especially in the rainy season. Loose soil that does not support strong constructions was considered a barrier for latrine use [56]. Although socio-cultural factors were mentioned in focus groups as potential barriers to latrine use, it was indicated that the practice was disappearing due to health education.

Results from the quantitative study indicated that characteristics of the house head (sex and age), household (income and size) and the latrine (bathing in the latrine and siting it on raised ground) were determinants of the intention to adapt the BVIP latrine to climate change. The study area for this work falls within the Zambezi valley which experiences frequent flooding and high air temperature (up to 40°C) in summer, particularly further north. Adapting the BVIP latrine to climate change by bathing in the latrine which is assumed to lower down latrine air temperature has consequential environmental implications. The BVIP latrine is a dry technology such that the addition of bathwater may pose operational challenges of odour control, potential groundwater contamination and pit filling.

Results from the focus groups indicate that most of the adaptation strategies of the BVIP latrine to climate change are central to the technology design. Addition of wood ash into the latrine pit to control odour appears a widely reported common practice [51]. Scientific empirical evidence for odour control using wood ash appears not readily available. Bathing in the latrine has potential impacts were discussed above. Barriers to climate change action in rural sanitation include the challenge to interface it with sanitation and hygiene programming (already complex) and that its data is perceived to be too confusing and discouraging to engage by practitioners [57]. This area still needs further research.

There is limited literature to discuss findings on sustained BVIP latrine use. Most studies in literature (i) are post-intervention evaluation studies, (ii) evaluate interventions at varying follow-up times in the post intervention period, (iii) use different sanitation options in interventions (at times unimproved, (iv) indicate participants were house heads or all household members, and (v) were done in different settings.

### Limitations of the study

The study potentially had interviewer-interviewee and researcher biases. Self-reported sanitation behaviour could have been over-reported. Local data collectors may know participants but could not be blinded. Interviewer-interviewee bias could have been avoided and/or minimised by (i) training data collectors, (ii) using pre-tested data collection instruments, (iii) review of the questionnaire by a WASH expert, (iv) administering the questionnaire in unannounced household visits and (v) physically checking on specified indicators of latrine use and characteristics using an inspection checklist. Potential researcher bias could have been avoided/minimised by triangulation in the mixed methods study [58]. It appears there is limited literature on the sustained use of a VIP latrine design outside intervention impact studies over long periods of time (e. g. 40 years) and using a standardised national sanitation option for fair comparison with the current results. This may limit the generalisation of the findings.

#### Policy implications and future research

Poor construction of BVIP latrines affects their operation for odour and fly control which in turn influence use. The long-observed unaffordability of the latrine design by poor households may indicate the need for speedy implementation of the new national sanitation policy draft to consider alternative options. However, it not certain how the identified factors influence sustained use of the BVIP latrine, which needs further scientific enquiry. There are opportunities to do a similar assessment in diverse rural settings with a wider selection of potential predictor variables which influence latrine use.

Policy implications of adapting sanitation to climate change may include the selection of appropriate technologies to help build resilience based on the existing experiences under specified contexts [22]. Modifying or extending the life of existing technologies may also adapt them to climate change to some extent [41]. Since households responded to climate change impacts on their sanitation facilities through some adaptation strategies, the provision of sanitation services in vulnerable rural areas may incorporate aspects of climate change. Further studies may be done to investigate household perceptions about climate change in vulnerable environments.

## Conclusions

The current study demonstrates high sustained use of the BVIP latrine, a national sanitation innovation for rural communities after four decades of implementation, not as a post-intervention evaluation study. Further, it shows a widening gap between local sanitation practice and review policy requirements, and the need to unlock the sanitation basket to allow for alternative options to address equity and universal access. The results show quite encouraging high sustained use of the BVIP latrine despite its perceived use barriers and low adoption due to unaffordability. The quantitative study shows that contextual factors were determinants of latrine use at the individual and household levels. Findings from the focus groups indicate that technology and social factors at the individual, household and community levels influence latrine use. Therefore, an interplay of multiple- level factors influence sustained latrine use. This is important as the country is about to consider other sanitation options. Climate change adaptation strategies that were implemented were central to the latrine design. They pose an extra cost to the capital requirements of constructing a BVIP latrine. There is need for community support in this respect. Alternative sanitation options and hygiene education may be needed to address unique household sanitation needs of a multicultural society in diverse environments and influence latrine use.

## Supporting information

S1 Fig Modified steps of the focus group discussion technique with permission [43]. (DOCX)

S1 Table Integrated behavioural model for water, sanitation and hygiene [30]. (DOCX)

S1 File Latrine use household questionnaire. (DOCX)

S2 File Informed consent document. (DOCX)

S3 File Blair ventilated improved pit latrine construction checklist. (DOCX)

S4 File Focus group discussion guide. (DOCX)

S5 File Phases of thematic analysis [46]. (DOCX)

S6 File Study data. (SAV)
