# Supplementary material for: Drivers and barriers to sustained use of Blair ventilated improved pit latrine after nearly four decades in rural Zimbabwe
Source: PLoS One. 2022 Apr 1;17(4):e0265077. doi: 10.1371/journal.pone.0265077 (PMC8975012; doi:10.1371/journal.pone.0265077)
Supplement: S1 Fig — (DOCX) [file pone.0265077.s001.docx]

**S1 Fig. Modified steps of the focus group discussion technique with permission [32]**

**RESULTS & REPORTING**

**ANALYSIS**

**RESEARCH DESIGN**

- **Study objectives**
- Purpose
- Schedule of questions
- Ethical clearance
- **Recruitment of participants**
- Group composition
- Number of participants
- Facilitator and assistant
- Number of focus groups
- **Venue**
- Accessible, no distractions
- Resources (recording, consent forms, first name tags)

**DATA COLLECTION**

- **Pre-session preparation**
- Familiarisation: group, equipment
- Recording
- **Familiarisation during meeting**
- Randomised self-introductions, consent, confidentiality, ground rules
- Discuss, record, observe, probe, reflect
- Tract questions for completeness, follow-up on discussion themes
- Conclude, acknowledge participants

***Thematic analysis [35]***
