# Supplementary material for: Drivers and barriers to sustained use of Blair ventilated improved pit latrine after nearly four decades in rural Zimbabwe
Source: PLoS One. 2022 Apr 1;17(4):e0265077. doi: 10.1371/journal.pone.0265077 (PMC8975012; doi:10.1371/journal.pone.0265077)
Supplement: S1 Table — (DOCX) [file pone.0265077.s002.docx]

**S1 Table. Integrated behavioural model for water, sanitation and hygiene [4]**

| Levels | Contextual factors | Psychosocial factors | Technology factors |
| --- | --- | --- | --- |
| Societal/Structural | Policy, climate, geography | Leadership, cultural identity | Manufacturing, financing, promotion and distribution of products |
| Community | Access to markets, access to resources, built and physical environment | Shared values, collective efficacy,  social integration, stigma | Location, access, availability, collective ownership, maintenance |
| Interpersonal/Household | Roles, household structure, division  of labour, available space | Norms, aspirations, shame, nurture | Access to product, demonstration of  use of products |
| Individual | Wealth, age, education, gender,  livelihoods | Self-efficacy, knowledge, disgust, perceived threat | Perceived cost, convenience,  strengths and weaknesses of product |
| Habitual | Facilitators/barriers to habit  formation | Existing water and sanitation  habits, outcome expectations | Ease and effectiveness of routine  use of product |
