# Supplementary material for: Drivers and barriers to sustained use of Blair ventilated improved pit latrine after nearly four decades in rural Zimbabwe
Source: PLoS One. 2022 Apr 1;17(4):e0265077. doi: 10.1371/journal.pone.0265077 (PMC8975012; doi:10.1371/journal.pone.0265077)
Supplement: S1 File — (DOCX) [file pone.0265077.s003.docx]

**S1 File. Latrine use household questionnaire**

No:

**Drivers and barriers to sustained use of the BVIP latrine, and its adaptation to climate change in rural Zimbabwe (Mbire district)**

Date …………………… Ward: ……… Village: ………………………………..……

*Note: Institutional and researcher details were purposively removed. This is used after*

*getting informed consent*

**Instructions to the enumerator**

- The questionnaire will only be administered after getting informed consent.
- Please do not prompt or read answers to the respondent, unless where it is indicated.
- This questionnaire is to be administered to the mother/female head of the household. If she is not available then you can administer it to male house head.
- Please circle one response and/or clearly write down responses where appropriate.

|  |  |
| --- | --- |
| 1. Sex | **1**. Male **2**. Female |
| 2. Marital status | **1**. Married **2**. Single |
| 3. Age group (years) | **1**. 18 – 25 **2**. 26 – 35 **3**. 36 – 45 **4**. 46 – 55  **5**. > 55 |
| 4. Educational level | **1**. No education **2**. Primary **3**. Secondary **4**. Tertiary |
| 5. Ethnicity | **1**. *Korekore*  **2.** *Chikunda*  **3**. Foreign **99**. Other  *If other, please specify: …………………………………….…* |
| 6. Religion | **1**. Christianity **2**. Traditional **3**. Muslim **4**. None **99.** Other  *If other, please specify*: ………………………………………. |
| 7. Monthly household  income /USD | **1**. Less than 50 **2**. 50 – 100 **3**. 101 – 200 **4**. Above 2000 |
| 8. Household size | **1**. ≤ 2 **2**. 3 – 5  **3**. > 5 |
| 9. Nature of household | **1**. Nucleus **2**. Extended |
| 10. No. of cattle owned | **1**. None  **2**. ≤ 3  **3**. 4 - 5 **4**. > 5 |
| 11. Residence period of  household (years) | **1**. < 1 **2**. 2 – 10 **3**. 11 - 20  **4**. > 20 |
| 12. Latrine option at the  household | **1**. uBVIP  **2.** BVIP |
| 13. Latrine has a clean  concrete slab | **1.** Yes  **2.** No |
| 14. Latrine has no bad  smell | **1**. Yes  **2**. No |
| 15. Latrine has a few  houseflies around it | **1**. Yes **2**. No |
| 16. Latrine interior is  dark | **1.** Yes  **2.** No |
| 17. Latrine is < 30 m  from the homestead | **1.** Yes  **2.** No |
| 18. Frequency of  latrine use (5 week  days) | **1.** Always/Usually used **2.** Sometimes used **3.** Never used |
| 19. How were  children’s (<5 years  old) stools disposed  of last time? | **1.** Children < 5 years old used latrine **2.** Put/rinsed into drain  **3**. Put/rinsed into latrine **4**. Thrown into garbage pit  **5**. Burried into ground **6**. Placed into open field  **7**. No children < 5 years old **99**. Other means  *If other, please specify* …………………………………………….. |
| 20. Main driver for use  of the BVIP latrine | **1.** Hygienic environment **2**. Latrine type  **3**. Easy to maintain (e. g. clean) **4**. Easily accessible  **5**. Health benefits **6**. Privacy and security  **99**. Other drivers  *If other, please specify* …………………………………………… |
| 21. Main barrier for  use the BVIP  latrine | **1.** Health risk exposure **2.** Design of the latrine  **3**. Risk of injury **4**. Unclean environment  **5**. > 30 m from the homestead **6**. Household social issues  **7**. None **99**. Other barriers  *If other, please specify* …………………………………………….. |
| 22. Do you intent to  adapt your BVIP  latrine to climate  change? | **1.** Yes **2**. No |
| 23. Reason for not  intending to adapt  your BVIP latrine to  climate change | **1**. No reason **2**. Induces extra cost **3**. Lacks knowledge  **4**. BVIP is strong **5**. Large family to bath  **5**. Unwilling to share with neighbours  **6**. Raised latrine affects accessibility **99**. Other reasons  *If other, please specify* ……………………………………………. |
| Would you adapt the BVIP latrine to climate change using the following strategies (24 – 31)?  24. Open defaecation? 1. Yes 2. No | |
| 25. Build a raised  latrine? | **1**. Yes **2.** No |
| 26. Build a standard  BVIP latrine? | **1**. Yes **2.** No |
| 27. Add wood ash to  the latrine pit? | **1**. Yes **2.** No |
| 28. Bath in the latrine? | **1**. Yes **2.** No |
| 29. Build latrine on  raised ground? | **1**. Yes **2.** No |
| 30. Build an emergency  latrine? | **1**. Yes **2.** No |
| 31. Share BVIP latrine  with neighbours | **1**. Yes  **2**. No |

**END OF THE QUESTIONNAIRE**

*Thank you for your participation*
