# Supplementary material for: Drivers and barriers to sustained use of Blair ventilated improved pit latrine after nearly four decades in rural Zimbabwe
Source: PLoS One. 2022 Apr 1;17(4):e0265077. doi: 10.1371/journal.pone.0265077 (PMC8975012; doi:10.1371/journal.pone.0265077)
Supplement: S2 File — (DOCX) [file pone.0265077.s004.docx]

**PARTICIPANT’S INFORMED CONSENT DOCUMENT**

**Drivers and barriers to sustained use of the BVIP latrine, and its adaptation to climate change in rural Zimbabwe (Mbire district)**

Principal investigator: [*Information was intentionally withheld*]

Contact details: *[Institutional details were intentionally withheld*]

Date: ……………………….. Time: ………………………

Dear prospective participant: …………………………………………………………..

Hello. My name is (*Interviewer name*). I am working with the Ministry of Health and Child Care district and provincial offices. You are invited to volunteer to participate in a research study for [*Institutional details removed*]. The purpose of the survey is to determine the drivers and barriers to sustained use of the BVIP latrine, and its adaptation to climate change in rural Zimbabwe (Mbire district). The initiative is develop a framework to select appropriate rural sanitation technology options. The final aim is to inform government policy to respond to global call to improve access to basic sanitation services for all in 2030.

Your participation is by responding to questions regarding yourself, household and sanitation facility. This is either at household level or through a small group where you meet your fellow community members to discuss the topic. If you have any questions you are free to ask. I can also refer you to our district office. Volunteering to participate will not expose you to any physical harm. However, if you feel you are not comfortable to respond to some of the questions you are free to indicate so.

Your name will not be used or connected to the information shared, recorded, presented at a conference or published. All data shared will be treated a s confidential. You will not be compensated for voluntarily participating in this study. Whatever is discussed in focus groups will have to be treated confidential by all members. During the interview I will also need your consent to inspect your sanitation facility (latrine) if available.

**Informed consent**

1. I confirm that I was told details of the study, how I should participate and understood it.

2. I was told of the risks or discomforts, and potential benefits of the study.

3. I was given adequate time to ask questions and I have no objections to participate.

4. I am aware that the information shared including personal details, will be anonymously

processed and presented when reporting the results.

5. I understand that I may withdraw my consent to participate any time without being affected

6. I understand and give my consent that focus group discussions will be audio-recorded.

7. I am participating willingly.

Participant …………………… OR put a cross (x) in the box Date: ……....………

^Signature^

Data collector: ………………………… Signature: ……………….. Date: ……………...

^Please print name^
