# Supplementary material for: Drivers and barriers to sustained use of Blair ventilated improved pit latrine after nearly four decades in rural Zimbabwe
Source: PLoS One. 2022 Apr 1;17(4):e0265077. doi: 10.1371/journal.pone.0265077 (PMC8975012; doi:10.1371/journal.pone.0265077)
Supplement: S3 File — (DOCX) [file pone.0265077.s005.docx]

**S3 Eile. Blair ventilated improved pit latrine construction check list**

**Drivers and barriers to sustained use of the BVIP latrine, and its adaptation to climate change in rural Zimbabwe (Mbire district)**

*[Institutional and researcher details were purposively removed]*

Introduction

Informed consent statement

Instruction: Please indicate your response for each question by putting a cross

|  | Checklist item | Response |
| --- | --- | --- |
| 1 | BVIP latrine constructed as an uBVIP design? | Yes/No |
| 2 | Latrine roof made of asbestos/iron sheets of concrete? | Yes/No |
| 3 | Superstructure made of fired bricks and cement? | Yes/No |
| 4 | Does the latrine have a vent pipe (brick/PVC)? | Yes/No |
| 5 | Does the vent pipe have a fly screen? | Yes/No |
| 6 | Does the latrine wall have cracks or holes? | Yes/No |
| 7 | Does the latrine have a concrete slab? | Yes/No |
| 8 | Is there a lid on the squat hole? | Yes/No |
| 9 | Description of the location of the latrine | High water table |
|  |  | Rocky/hilly |
|  |  | Densely vegetated |
|  |  | Sandy soil |
|  |  | Loamy/clayey soil |
|  |  | Other |
