# Supplementary material for: Drivers and barriers to sustained use of Blair ventilated improved pit latrine after nearly four decades in rural Zimbabwe
Source: PLoS One. 2022 Apr 1;17(4):e0265077. doi: 10.1371/journal.pone.0265077 (PMC8975012; doi:10.1371/journal.pone.0265077)
Supplement: S4 File — (DOCX) [file pone.0265077.s006.docx]

**S4 File. Focus group discussion guide**

*[Institutional and researcher details were purposively removed]*

**Drivers and barriers to sustained use of the BVIP latrine, and its adaptation to climate change in rural Zimbabwe (Mbire district)**

**1. Introduction**

1.2. Self-introduction (facilitator) and assistant: Thanking participants for coming

1.3. Introduction of the study:

(a) Purpose and how participants were recruited

(b) Expected duration, use of information collected

(c) Ground rules: how participants will response, no wrong/correct answers

(d) Discussions will be audio-recorded

1.4 Self-introductions of participants: First names basis only

1.5 Informed consent: Voluntary participation, confidentiality, anonymity, right to withdraw,

how to disclose findings (publications)

1.6 Asking for clarifications or any other questions

1.7. Signing of consent form/verbal agreement to participate

1.8. Writing of name cards (first name basis)

1.9. Filling in short demographic data: Sex, age group, highest level of education reached,

marital status, community leader/worker, professional qualification.

2. Discussions

*2.1 Question 1: What are the drivers for you to use the BVIP latrine?*

Facilitator starts by asking general knowledge about the BVIP latrine to make respondents comfortable (What is it? How is it built? How does it work? e. t. c) …

At the end facilitator summarises what participants said and adds on to it where necessary.

1. *Drivers*
2. *Barriers*

Facilitator controls the discussions, reflecting probing, asking participant experiences, opinions, beliefs where necessary.

When participants feel they have exhausted the question, the facilitator may ask for clarifications, any other contributions or questions, and summarises to make sure participants agree

*2.2. Question 2: How do households adapt the BVIP latrine to climate change?*

Facilitator explains climate change and asks participants to give examples and potential risks related to sanitation.

Group discussion (as in question 1)

2.3. Closure: At the end, the facilitator thanks the participants and they depart.

Supplementary file 5. Modified flow chart of the steps of the focus group discussion technique with permission [32]
