# Supplementary material for: Drivers and barriers to sustained use of Blair ventilated improved pit latrine after nearly four decades in rural Zimbabwe
Source: PLoS One. 2022 Apr 1;17(4):e0265077. doi: 10.1371/journal.pone.0265077 (PMC8975012; doi:10.1371/journal.pone.0265077)
Supplement: S5 File — (DOCX) [file pone.0265077.s007.docx]

**S5 File. Table. Phases of thematic analysis [36]**

|  | Phase | Description of the process |
| --- | --- | --- |
| 1 | Familiarizing yourself with  your data: | Transcribing data (if necessary), reading and re-reading the data, noting down initial ideas. |
| 2 | Generating initial codes: | Coding interesting features of the data in a systematic fashion across the entire data set, collating data relevant to each code. |
| 3 | Searching for themes: | Collating codes into potential themes, gathering all data relevant to each potential theme. |
| 4 | Reviewing themes: | Checking if the themes work in relation to the coded extracts (Level 1) and the entire data set (Level 2), generating a thematic ‘map’ of the analysis. |
| 5 | Defining and naming  themes: | Ongoing analysis to refine the specifics of each theme, and the overall story the analysis tells, generating clear definitions and names for each theme. |
| 6 | Producing the report: | The final opportunity for analysis. Selection of vivid, compelling extract examples, final analysis of selected extracts, relating back of the analysis to the research question and literature, producing a scholarly report of the analysis. |
